# Palliative Care Consultation and Effect on Length of Stay in a Tertiary-Level Neurological Intensive Care Unit

**DOI:** 10.1089/pmr.2020.0051

**Published:** 2020-08-14

**Authors:** Michael Pottash, Danielle McCamey, Hunter Groninger, Edward F. Aulisi, Jason J. Chang

**Affiliations:** ^1^ Division of Palliative Medicine, Department of Medicine, MedStar Washington Hospital Center, Washington, DC, USA.; ^2^ Department of Medicine, Georgetown University School of Medicine, Washington, DC, USA.; ^3^ Department of Critical Care and Preanesthesia, MedStar Washington Hospital Center, Washington, DC, USA.; ^4^ Department of Neurosurgery, MedStar Washington Hospital Center, Washington, DC, USA.; ^5^ Department of Critical Care Medicine, MedStar Washington Hospital Center, Washington, DC, USA.; ^6^ Department of Neurology, Georgetown University School of Medicine, Washington, DC, USA.

**Keywords:** brain injury, cerebrovascular accident, neurocritical care, neurosurgery, palliative care, palliative medicine

## Abstract

***Background:*** Patients admitted to an acute care setting with a devastating brain injury are at high risk for morbidity and mortality. These patients and their families can benefit from the psychosocial and decision-making support of a palliative care consultation.

***Objective:*** We aim to investigate the characteristics and impact of palliative care consultation for patients under the management of neurosurgical and critical care services with a devastating brain injury in a neurological intensive care unit (ICU) at a large tertiary-care hospital.

***Design:*** Data were collected by retrospective review of the electronic medical record and metrics collected by the palliative care service. Data were analyzed using descriptive statistics. Linear regression analysis was performed to assess effect of timing of palliative care consultation.

***Results:*** Fifty-five patients admitted to the neurological ICU under the management of the neurosurgical service received a palliative care consultation for the following: hemorrhagic stroke (49%), metastatic cancer (22%), and traumatic brain injury (18%). Of these, 73% had at least one neurosurgical intervention. Palliative care was most frequently consulted for assistance in defining a patient's goals of care (88%). When compared with late consultation, early palliative care consultation was significantly associated with shorter mean length of stay (LOS) and positively correlated in linear regression analysis without an effect on mortality.

***Conclusions:*** When compared with a late consultation, early palliative care consultation corresponded to shorter LOS without increasing mortality. One reason for this effect may be that palliative care can help to clarify and document goals of care earlier and more concretely.

## Introduction

Patients suffering from devastating brain injuries (DBI)—severe traumatic brain injury, ruptured aneurysmal subarachnoid hemorrhage, intracerebral hemorrhage, and acute ischemic stroke—are at high risk for morbidity and mortality, are often subject to unclear prognostication, and are frequently unable to participate in shared medical decision-making, placing considerable strain on distressed families.^1,2^ For these reasons, palliative care plays an important part in providing high-quality care to patients with DBI who are admitted to a neurological intensive care unit (ICU).^3^

For critically ill patients, palliative care has been shown to improve communication of prognosis, and interventions focus on eliciting patient and family values, helping families understand options, and mitigating conflict.^4,5^ In addition, there is growing evidence that palliative care consultation can decrease ICU length of stay (LOS) without affecting mortality.^5–7^

Notably, current literature rarely distinguishes the specific palliative care needs of patients with DBI who require neurosurgical care. Patients who require management by a neurosurgeon tend to have more extensive neurological injury and a poorer prognosis.^3^ To provide high-quality palliative care, palliative care specialists must appreciate the distinct culture of neurosurgery and the unique relationship that is formed between patient/surrogate decision-maker and surgeon.^8^

To provide the highest quality palliative care to patients who suffer from DBI, we sought to delineate the nature of palliative care consultation for patients with DBI admitted to a level-one tertiary-care trauma center. We described the characteristics and outcomes of patients with DBI who received palliative care consultation, and we described how the timing of palliative care consultations may impact various outcomes such as hospital LOS and mortality in patients under neurosurgical care in a neurological ICU.

## Methods

After institutional review board approval, we performed a retrospective review of the electronic medical record and metrics collected by the palliative care service for all patients in a one-year period who received a palliative care consultation while admitted to a neurological ICU under the management of neurosurgical and critical care services. MedStar Washington Hospital Center is a 912-bed tertiary-care level-one trauma center located in Washington, D.C. that includes a designated 14-bed neurological ICU. In the neurological ICU, the neurosurgery team serves as the primary team with critical care physicians as consultants.

At our institution, palliative care consultation requires an order from the attending physician of record. Once a consult request is received, palliative care providers collaborate with the primary team to determine the specific reason(s) for consultation and shared clinical goals. The palliative care team is multidisciplinary and consists of a medical provider (physician and/or nurse practitioner) and psychosocial-spiritual provider (social worker and/or chaplain).

Surgical procedures were considered any invasive bedside or operative procedure(s), including external ventricular drains, craniotomy and resection, decompressive hemicraniectomy, spinal procedures, and endovascular procedures (coiling and embolization). In our health system “late palliative care consultation” is defined as any request for consultation made after the fourth day of hospitalization and is based on data showing cost savings with earlier consultation.^9^

### Statistical analysis

Initial demographic variables and outcome variables were descriptively evaluated. In addition, a comparison of demographic variables between patients who received early and late palliative care consults were evaluated with independent samples *t*-test and chi-square test. Kolmogorov–Smirnov goodness-of-fit test was used to evaluate for normal distribution of continuous variables. LOS was not found to have a normal distribution (*p* < 0.001 by Kolmorogov–Smirnov test) and was natural logarithm transformed to satisfy the Kolmogorov–Smirnov goodness-of-fit test (*p* = 0.200).

The natural logarithm of LOS was evaluated as the dependent variable in simple and multiple linear regression models evaluating independent associations between baseline characteristics and LOS. A *p*-value of <0.05 was selected as a cutoff in simple linear regression analyses for inclusion in the multiple linear regression model. Predictor variables that were significant at *p* < 0.05 were retained in the multiple linear regression model. Associations were presented as unstandardized linear regression coefficients with corresponding confidence interval (95% CIs). The Statistical Package for Social Science (version 25.0 for Windows; SPSS Inc., Chicago, IL) was used for statistical analyses.

## Results

In 2017, 1095 patients were admitted to the neurocritical care under the management of the neurosurgical service. Of these patients, 55 patients received a palliative care consultation. Demographics for patients seen by palliative care are described in Table 1.

**Table 1.** Baseline Characteristics for Patients Who Received Palliative Care Consults in the Neurosurgical Intensive Care Unit

Demographic variables	N
Male gender (%)	25 (45)
Ethnicity (%)	
African American (%)	35 (64)
White (%)	15 (27)
Other (%)	3 (5)
Asian (%)	1 (2)
Unknown (%)	1 (2)
Transfer from outside hospital (%)	27 (49)
PMH	
Cancer (%)	19 (35)
Stroke (%)	9 (16)
End-stage renal disease (%)	5 (9)
Dementia (%)	2 (4)
Congestive heart failure (%)	2 (4)
Pulmonary hypertension (%)	1 (2)
Chronic obstructive pulmonary disease (%)	1 (2)
Coronary artery disease (%)	1 (2)
Diagnosis	
Hemorrhagic stroke (intracerebral hemorrhage, intraventricular hemorrhage, subarachnoid hemorrhage) (%)	27 (49)
Cancer (%)	12 (22)
Traumatic brain injury (subdural hemorrhage, gunshot wound to the brain) (%)	10 (18)
Spinal cord injury (%)	4 (7)
Infected ventriculoperitoneal shunt (%)	1 (2)
Hydrocephalus (%)	1 (2)
Surgical intervention (%)^a^	40 (73)

^a^ Surgical intervention = decompressive hemicraniectomy, tumor resection, cerebrovascular procedure, spinal surgery, abscess drainage, ventriculoperitoneal shunt revision, external ventricular drain.

PMH, past medical history.

The most common admission diagnoses of patients seen by palliative care were hemorrhagic stroke (49%), followed by malignancy (22%), and traumatic brain injury (18%). Of these patients, 73% had at least one neurosurgical intervention performed during their admission.

Reasons for consulting palliative care and palliative care interventions are listed in Table 2. Most frequently, palliative care was consulted for assistance in better defining a patient's goals of care (88%). Although 65% of these patients died during hospitalization or were discharged to hospice, about one-third were discharged with the intention to rehabilitate. The mean time to palliative care consultation was 10 days; 62% of consults were requested after 4 days.

**Table 2.** Outcomes for Patients with Palliative Care Consults in the Neurosurgical Intensive Care Unit

Outcome	N
Length of stay, mean (SD), days	20 (19)
Disposition	
Home (%)	4 (7%)
Rehab (%)	2 (4%)
Nursing home	10 (18%)
Long-term acute care hospital (%)	2 (4%)
Hospice (%)	9 (16%)
Died during hospitalization (%)	28 (49%)
Mean time to palliative care consultation (days)	10
Late palliative care consult (%)^a^	34 (62%)
Reason for palliative care consult	
Goals of care (%)	50 (88%)
Pain (%)	5 (9%)
Nonpain symptoms (%)	6 (11%)

^a^ Late palliative care consult = palliative care consult made after four days of hospitalization.

Table 3 summarizes demographic and outcome variables for those patients who received early versus late palliative care consultations. When compared with late consultation, early palliative care consultations were significantly associated with older mean age (69 years vs. 65 years old, *p* = 0.024), shorter mean LOS (8 days vs. 28 days, *p* = 0.001), absence of a neurosurgical intervention (23% vs. 77%, *p* < 0.001), more frequent past medical history (PMH) of cancer (58% vs. 42%, *p* = 0.029), more frequent PMH of end-stage renal disease (80% vs. 20%, *p* = 0.044), more frequent occurrence of brain tumor as the admitting diagnosis (67% vs. 33%, *p* = 0.022), and less frequent occurrence of traumatic brain injury as the admitting diagnosis (0% vs. 100%, *p* = 0.016). No association was found between timing of palliative care consultations and mortality.

**Table 3.** Comparison of Demographics Based on Early versus Late Palliative Care Consultation

Baseline characteristic	Early palliative care consult	Late palliative care consult^a^	p
Mean age (years, SD)	69 (11.28)	65 (18.46)	0.024
Length of stay (days, SD)	8.1 (6.48)	28.0 (20.21)	0.001
Hospital mortality	43%	57%	0.467
Male gender	32%	68%	0.389
African American race	29%	71%	0.052
Surgical intervention^b^	23%	77%	<0.001
Transferred from outside hospital	48%	52%	0.135
PMH cancer	58%	42%	0.029
PMH stroke	22%	78%	0.281
PMH end-stage renal disease	80%	20%	0.044
PMH dementia	0%	100%	0.258
PMH congestive heart failure	50%	50%	0.726
PMH pulmonary HTN	0%	100%	0.428
PMH chronic obstructive pulmonary disease	0%	100%	0.428
PMH coronary artery disease	0%	100%	0.428
Admission hemorrhagic stroke	41%	59%	0.701
Admission spinal cord injury	0%	100%	0.103
Admission brain tumor	67%	33%	0.022
Admission traumatic brain injury	0%	100%	0.016

^a^ Late palliative care consult = palliative care consult made after four days of hospitalization

^b^ Surgical intervention = decompressive hemicraniectomy, tumor resection, cerebrovascular procedure, spinal surgery, abscess drainage, ventriculoperitoneal shunt revision, external ventricular drain.

Table 4 shows associations between baseline characteristics and the natural log of LOS (lnLOS) in simple and multiple linear regression models. lnLOS was associated with the following variables in simple linear regression analysis: age (*p* = 0.040), surgical intervention (*p* = 0.002), admission diagnosis of traumatic brain injury (*p* = 0.041), and timing of palliative care consultation (*p* < 0.001). In multiple linear regression analysis, timing of palliative care consultation was independently and positively associated (*p* < 0.001) with LOS (linear regression coefficient 1.138, 95% CI 0.603–1.673). Age was also found to be independently and negatively associated (*p* = 0.045) with LOS (linear regression coefficient −0.014, 95% CI −0.027 to 0.000).

**Table 4.** Simple and Multiple Linear Regression Analyses between Baseline Characteristics and Natural Log of Length of Stay

Variable	Simple linear regression analysis		Multiple linear regression analysis	
	Linear regression coefficient (95% CI)	p	Linear regression coefficient (95% CI)	p
Age	−0.0180 (−0.0350 to 0.001)	0.040	−0.014 (−0.027 to 0.000)	0.045
Male gender	0.339 (−0.216 to 0.895)	0.226		
African American race	0.510 (−0.056 to 1.076)	0.077		
Surgical intervention^a^	0.932 (0.357 to 1.507)	0.002	0.261 (−0.294 to 0.816)	0.350
Transfer from outside hospital	−0.497 (−1.041 to 0.0470)	0.072		
PMH cancer	−0.327 (−0.910 to 0.256)	0.265		
PMH stroke	0.703 (−0.030 to 1.436)	0.060		
PMH end-stage renal disease	−0.644 (−1.603 to 0.316)	0.184		
PMH dementia	−0.282 (−1.779 to 1.214)	0.707		
PMH congestive heart failure	−0.225 (−1.722 to 1.272)	0.765		
PMH pulmonary hypertension	−0.395 (−2.491 to 1.702)	0.707		
PMH chronic obstructive pulmonary disease	0.604 (−1.488 to 2.697)	0.565		
PMH coronary artery disease	0.311 (−1.786 to 2.409)	0.767		
Admission hemorrhagic stroke	−0.262 (−0.818 to 0.295)	0.350		
Admission spinal cord injury	0.725 (−0.337 to 1.786)	0.177		
Admission brain tumor	−0.347 (−1.019 to 0.325)	0.305		
Admission traumatic brain injury	0.797 (0.0320 to 1.561)	0.041	0.196 (−0.294 to 0.816)	0.553
Late palliative care consult^b^	1.361 (0.922 to 1.800)	<0.001	1.138 (0.603 to 1.673)	<0.001

^a^ Surgical intervention = decompressive hemicraniectomy, tumor resection, cerebrovascular procedure, spinal surgery, abscess drainage, ventriculoperitoneal shunt revision, external ventricular drain.

^b^ Late palliative care consult = palliative care consultation requested after four days of hospitalization.

CI, confidence interval.

## Discussion

In this study, simple and multiple linear regression models demonstrate that LOS was significantly shorter when patients received an early palliative care consultation when compared with a late consultation. This important finding reflects the growing body of literature that suggests earlier palliative care consultation may correspond to decreased LOS, especially in ICU patients.^10^ Although a common perception is that earlier palliative care consultation decreases LOS by guiding family or legal representatives to withdraw of life-sustaining therapy earlier in the hospital course, chi-square analysis (Table 2) showed that hospital mortality was not associated with early or late palliative care consultation. To our knowledge, this study is the first to demonstrate this association, specifically in the neurosurgical population.

One mechanism by which timing of palliative care consultation may affect hospital LOS is by defining and documenting goals of care earlier and more concretely. This is reflected in our finding that the most common reason for palliative care consultation was to assist the neurosurgical team in conferencing with families to help clarify goals of care.

The majority of our patients who received a palliative care consultation had a neurosurgical intervention. This was unexpected, since the literature describes the unique bond that is formed in the process of intervention wherein the surgeon receives buy-in from the patient and is less inclined to include outsiders in the care of their patients.^8,11^ We did find that surgical intervention was associated with later palliative care consultation, which is consistent with conventional wisdom. However, surgical intervention was not a significant predictor for longer LOS in multiple linear regression analysis.

We found several other trends of note. As with patients undergoing a neurosurgical intervention, admission for traumatic brain injury was associated with a later palliative care consultation. Furthermore, admission for a diagnosis of cancer/brain tumor was associated with an earlier palliative care consultation, perhaps related to greater prognostic certainty.

Our research has several limitations. First, this study was retrospective in nature with a modest sample size, and a nonrandomized patient selection. This may have led to selection bias, in particular, as palliative care consultation often occurred late—as in this cohort—and presumably when the treating teams felt that they had exhausted all interventions.

Second, the identification of “late” as a palliative care consult after four days may represent an arbitrary dividing line between obtaining palliative care consultation too early or too late. Often, ongoing emergent interventions—such as intracranial pressure management—may require multiple days or even weeks of therapy. In such cases, it is reasonable to question the optimal time for a palliative care consultation.

Although there are significant limitations, we believe that this trend is worthy of further investigation to understand whether timing of palliative care consultation can truly affect hospital LOS and by what mechanism.

## Conclusion

In this single-center study, we found that when compared with a late consultation, early palliative care consultation may be an independent predictor for shorter LOS, independent of mortality. These observations require independent confirmation in other institutions and further studies to better differentiate reasons for this observation.

## Abbreviations Used

DBI: devastating brain injuries
ICU: intensive care unit
LOS: length of stay
PMH: past medical history
TBI: -
